# Ibuprofen, Flurbiprofen or Naproxen Sodium Minimally Influences Musculoskeletal Adaptations to Treadmill Exercise in Rats

**DOI:** 10.1002/jcsm.13798

**Published:** 2025-04-10

**Authors:** Brandon M. Roberts, Alyssa V. Geddis, Alexandra Ciuciu, Marinaliz Reynoso, Nikhil Mehta, Alyssa N. Varanoske, Alyssa M. Kelley, Maximus C. Leiss, Alexander L. Kolb, Julie M. Hughes, Marshall A. Naimo, Ryan E. Tomlinson, Jeffery S. Staab

**Affiliations:** ^1^ Military Performance Division US Army Research Institute of Environmental Medicine Natick Massachusetts USA; ^2^ Sargent College of Health & Rehabilitation Sciences Boston University Boston Massachusetts USA; ^3^ Department of Orthopaedic Surgery Thomas Jefferson University Philadelphia Pennsylvania USA; ^4^ Oak Ridge Institute for Science and Education Belcamp Maryland USA

**Keywords:** bone, COX inhibitor, COX‐1, COX‐2, mechanical loading, skeletal muscle

## Abstract

**Background:**

Non‐steroidal anti‐inflammatory drugs (NSAIDs) may influence musculoskeletal health. The purpose of this study was to compare the effects of three different NSAIDS: naproxen sodium, ibuprofen, flurbiprofen or a placebo on musculoskeletal adaptations in rodents with or without 6 weeks of aerobic exercise.

**Methods:**

Nine‐week‐old male Wistar rats (*n* = 80) were randomized to either exercise (EX) or no‐exercise control (CON) conditions and treated with naproxen, ibuprofen (IBU), flurbiprofen (FLU) or placebo (PLA). For exercise, rats ran 5 days per week for 6 weeks at a 5% incline on a motorized treadmill for 30 min. Three‐point bending (3 PB) and microcomputed tomography (microCT) were measured in the femur. Anabolic muscle signalling pathways were measured in the quadriceps. Muscle fibre cross‐sectional area (CSA) and fibre type were measured in the soleus. Data were analysed using a two‐way ANOVA for treatment by condition and is visualized as mean ± standard deviation.

**Results:**

For 3 PB, there was an exercise effect for ultimate bending energy, postyield energy, toughness, postyield toughness, postyield displacement, ultimate strain and postyield strain (all, *p* < 0.05). There was a treatment by condition effect for Young's Modulus, where placebo exercise was less than placebo control (PLA EX: 3256.44 ± 463.41 MPa, PLA CON: 4849.94 ± 836.70 MPa, *p* < 0.05). For microCT, there was a treatment by condition effect for trabecular thickness (*p* = 0.047) and the IBU EX group increased thickness compared with the IBU CON group (IBU EX: 0.133 ± 0.011 mm, IBU CON: 0.121 ± 0.007 mm, *p* = 0.027). In the quadriceps, for myosin heavy chain abundance, there was a treatment by condition effect (*p* = 0.046) and ibuprofen exercise was lower than ibuprofen control (IBU EX: 0.636 ± 0.513 AU, IBU CON: 1.81 ± 1.012 AU, *p* = 0.016). There was no treatment by condition effect for phosphorylation of the AKT, AMPK or ERK pathways (all, *p* > 0.05). In the soleus, there was no treatment by condition effect for fibre type percentage or muscle CSA (*p* > 0.05).

**Conclusions:**

NSAIDs did not have a strong negative or positive effect on musculoskeletal adaptations to 6 weeks of treadmill running in young healthy male rodents.

## 1 Introduction

Non‐steroidal anti‐inflammatory drugs (NSAIDs) are widely used in combination with exercise and inhibit cyclooxygenase (COX) enzymes [1, 2]. In muscle and bone, COX‐1 is constitutively expressed and is primarily involved in tissue homeostasis, and COX‐2 abundance is low or absent until perturbed by exercise or injury [3]. During exercise, arachidonic acid (AA) is released from membrane phospholipids, which is then converted to prostaglandins by COX enzymes. NSAIDs inhibit COX enzymes, blocking the production of prostaglandins, thereby potentially limiting adaptations to exercise [4]. NSAIDs can be classified by their relative degree of COX selectivity towards COX‐1 or COX‐2 [5]. For example, naproxen sodium and ibuprofen have COX‐1/COX‐2 selectivity ratios of 3.8 and 2.6, respectively, whereas the NSAID flurbiprofen has a ratio of 10 [5].

Previous research has demonstrated that taking NSAIDs before or after exercise influences musculoskeletal adaptations [6]. Specifically, COX‐2 regulates muscle regeneration and NSAIDs selective for COX‐2 can attenuate regrowth of myofibers [7]. COX‐2 inhibition also reduces myofiber growth in both slow‐twitch and fast‐twitch muscle fibres after a localized freeze injury [8]. Previous studies have also shown ibuprofen reduces muscle hypertrophy with synergistic ablation and in response to running in rodents [9, 10]. Furthermore, in vitro models have found detrimental effects of NSAIDs on skeletal muscle at varying concentrations [11, 12]. However, a current gap in the current literature exists where endurance‐based exercise and physiological doses of NSAIDs of varying selectivity have not been tested simultaneously against a placebo. This is important to better understand how commonly used NSAIDs might affect muscle adaptations to endurance exercise, particularly at lower doses that reflect human use, and to determine whether specific NSAIDs have unique or differential impacts on musculoskeletal health.

Bone responds to loading activity through the processes of adaptive bone formation and resorption remodelling where areas of bone microdamage are resorbed and replaced with new bone tissue, improving resistance to fatigue and fracture [13]. Prostaglandin signalling is thought to be an important mediator of these processes and can be inhibited by NSAIDs [14]. Indeed, the effects of NSAIDs on bone remodelling and healing are mixed in the literature [15]. For example, indomethacin, a strong COX‐1 inhibitor, inhibits the bone healing process in animal models of bone trauma [16, 17, 18]. Conversely, flurbiprofen, another COX‐1 inhibitor, may improve skeletal adaptations in growing rats [19]. Naproxen sodium and celecoxib, strong COX‐2 inhibitors, impair bone formation in a repetitive axial forelimb compression in mice [20] and may impair fracture healing in rats [21]. However, in vitro data suggest that therapeutically relevant concentrations do not exert any negative influence on cell growth and proliferation in osteogenic differentiated stem cells [22]. Overall, these findings highlight the complex and context‐dependent effects of NSAIDs on bone and the need to test them in less extreme models.

The purpose of this study was to compare the effects of three NSAIDs having a wide range of COX selectivity, compared with a placebo, with or without endurance‐based treadmill exercise, on musculoskeletal health in rats at doses below the no‐observed‐adverse‐effect‐level (NOAEL). We hypothesized that naproxen sodium and ibuprofen would inhibit exercise‐induced bone formation, but flurbiprofen would improve it, due to COX selectivity differences. We also hypothesized that NSAIDs would attenuate the acute muscle signalling to treadmill running and result in differences in chronic skeletal muscle changes in myosin isoforms and muscle size.

## 2 Methods

All animal care and procedures were approved by the Institutional Animal Care and Use Committee of the US Army Research Institute of Environmental Medicine (USARIEM) in accordance with the National Institutes of Health Guide for the Care and Use of Laboratory Animals. USARIEM is accredited by the Association for Assessment and Accreditation of Laboratory Animal Care. Male adult Wistar rats (*n* = 80) were purchased weighing 175 g and received at ~8–9 weeks of age, were acclimated for 2 weeks prior to experiments and were 16–17 weeks old at an endpoint, which is comparable with a ~20–21‐year‐old adult. Animals were housed in individual cages on a 12:12 light/dark cycle, at a temperature of 22°C–23°C with running wheels and allowed ad libitum access to food and water. Prior to experiments, animals were block randomized to one of two conditions: exercise or control, and one of four treatment groups (placebo, naproxen, ibuprofen and flurbiprofen). Body weight, wheel running activity, food and water intake were recorded daily.

### 2.1 Exercise

After a 1‐week treadmill running familiarization period, the exercise groups ran 5 days per week for 30 min per day for 6 weeks. The protocol consisted of 2 min at 15 cm/s (9 m/min), 2 min at 20 cm/s (12 m/min) and 26 min at 25 cm/s (15 m/min), all at a 5% incline (Harvard Apparatus, Holliston, MA). Control animals were placed on a stationary treadmill for an equivalent time. To motivate animals to run on the treadmill, mild stimuli were applied, including either a gentle shock from an electric grid at the back of the treadmill (0.2 mA) or intermittent air puffs directed at their hindquarters. When an animal received a cumulative shock of 30 s in one running session, the shock stimulus was stopped and air puffs were used. After the 6‐week exercise or control period, rodents were rapidly anaesthetised and euthanized 20–30 min after their final exercise bout or control procedures. Skeletal muscle was harvested, snap froze in liquid nitrogen or cooled in isopentane and stored at −80°C until analysed. Femurs were dissected, allowed to come to room temperature (RT) and then placed in PBS‐soaked gauze and stored at −20°C until analysed.

### 2.2 NSAID Administration

NSAIDs were administered through drinking water, starting a day before the training program commenced, similar to previous studies [7, 9, 10, 23]. The dosage for each NSAID was determined based on the maximum human equivalent dose for 65 kg adult as previously described [23], while staying under the NOAEL for rats, which is 40 mg/kg/day for naproxen sodium, 200 mg/kg/day for ibuprofen and 50 mg/kg/day for flurbiprofen. Groups were dosed with NSAIDs relative to their body weight at the beginning of each week with one of the following: 20 mg/kg of naproxen sodium, 200 mg/kg of ibuprofen or 25 mg/kg of flurbiprofen. NSAIDs were mixed into 400 mL of the drinking water and refreshed two to four times per week, whereas the placebo group received the same volume of water without NSAIDs.

### 2.3 Microcomputed Tomography

To determine bone mass and geometry, each bone was scanned using a Bruker Skyscan 1275 microCT system. Briefly, each bone was scanned individually using a 1‐mm aluminium filter and scan settings of 55 kV, 181 μA and 74 ms of exposure. The long axis of the bone was parallel to the *z*‐axis of the scanner to obtain transverse scan slices with an isometric voxel size of 13 μm. Images were reconstructed using nRecon (Bruker) and analysed using CTan (Bruker).

### 2.4 Three‐Point Bending

After microCT scanning, structural and mechanical properties of the femur were quantified using standard 3‐point bending (3 PB) to obtain force–displacement curves, and the force–displacement curves were converted to stress–strain using microCT‐based geometry and analysed using a custom GNU Octave script.

### 2.5 Nuclear Magnetic Resonance

Nuclear Magnetic Resonance Imaging (NMR, Bruker Biospin Corporation, Billerica, MA) was utilized to measure body composition (lean mass and fat mass). Body composition was measured at baseline and endpoint.

### 2.6 Protein Extraction and Immunoblotting

Protein was extracted from the quadricep and soleus. Fifteen to 20 mg of muscle was cut and placed into 1× Lysis buffer and Halt protease/phosphatase inhibitor cocktail (#78446, Cell Signaling Technologies, Danvers, MA, USA) and homogenized using a Bead Ruptor Elite homogenizer (#19‐040E, Omni International, Kennesaw, GA, USA). The homogenate was centrifuged at 12 000 g for 10 min at 4°C, and the subsequent supernatant was collected and quantified by Bradford Assay to be used in western immunoblotting. Protein samples were run in 12‐well 4%–20% tris‐glycine gels (#XP04202BOX, Thermofisher Scientific, Waltham, MA, USA) for ~1.5 h at 125 V. Gels were transferred for 21–24 h at 27 V; once removed from transfer, the membranes were washed in 1× tris buffered saline with tween (TBST) once for 5 min and then subsequently blocked in 5% non‐fat dry milk (#1706404XTU, Thermofisher Scientific) for at least 1 h. Membranes were then washed in 1× TBST three times for at least 5 min each wash. Membranes were then incubated in primary antibody dilutes in 5% BSA and TBST overnight at 4°C on a tube rotator in the following antibodies: p‐AKT S473 (#9271), Total AKT (#9272), p‐AMPKα T172 (#2535), Total AMPK α (#2603), p‐S6 S235/236 (#4858), p‐S6 S240/244 (#2215), Total S6 (#2217), p‐ERK 42/44 (#9101), Total ERK (#9102), GAPDH (#2118), p‐4EBP1 S65 (#9451), Total 4EBP1 (#9644, all antibodies to this point from Cell Signaling Technologies), myosin heavy chain fast (#M1570, Sigma Aldrich), myosin heavy chain slow (#ab11083) or total myosin heavy chain (#MAB4470, R&D Systems, Minneapolis, MN, USA). Membranes were then incubated in either anti‐rabbit IgG antibody (#7074S) or anti‐mouse IgG antibody (#7076, Thermofisher Scientific) secondary antibody and imaged using a Chemidoc XRS+ (#12003153, Biorad Hercules, CA, USA). Densiometric quantification analysis was done using NIH Image J 1.60.

## 3 Muscle Fibre Cross‐Sectional Area

For muscle histology, the soleus muscle was embedded in optimal cutting temperature compound after dissection (#4583, Sakura Finetek, Torrance, CA, USA) and quickly frozen in isopentane cooled by liquid nitrogen for 15–30 s and then stored at −80°C. The frozen muscle was sliced into sections of 6–8 μm using a microtome cryostat (Leica Biosystems, Deer Park, IL, USA). Sections were fixed in 4% paraformaldehyde for 20 min at RT and then blocked for 120 min in a buffer containing 5% goat serum (#5425S), 2% BSA (#A3059, Cell Signaling Technologies Danvers, MA, USA) and 0.5% Triton (#X‐100, Sigma Aldrich, St. Louis, MO, USA). Sections were rinsed twice in phosphate‐buffered saline (PBS) and blocked again with 5% goat serum at RT. Next, sections were incubated with myosin heavy chain I antibody (#BA‐D5, Developmental Studies Hybridoma Bank, Iowa City, IA, USA) at a 1:100 concentration in 1% goat serum and PBS for 30 min at 37°C followed by three, 5‐min PBS washes at RT. The slices were then incubated in Alexa 647 GAM antibody (#A21236, Thermofisher Scientific, Waltham, MA, USA) at a 1:200 concentration in 1% goat serum in PBS for 30 min at 37°C, followed by three, 5‐min PBS washes at RT. Afterwards, sections were blocked with 5% goat serum for another 30 min at RT, rinsed twice with PBS and incubated with laminin antibody (#L9393, Sigma Aldrich, at a 1:50 concentration in 1% goat serum for 30 min at RT, followed by Alexa 405 (1:200 w/v in 1% goat serum, #L9393, Thermofisher Scientific) for 30 min. Next, a myosin heavy chain II (#SC‐71, Developmental Studies Hybridoma Bank) antibody was applied to the sections at a concentration of 1:80 in 1% goat serum in PBS for 30 min at 37°C, followed by three, 5‐min PBS washes at RT. Lastly, the sections were incubated in Alexa 488 GAM (#A‐21121, Thermofisher Scientific) antibody at a concentration of 1:200 in 1% goat serum in PBS for 30 min at 37°C, followed by a final three, 5‐min PBS washes at RT. A coverslip was then mounted on each slide using prolong (#P10144, Thermofisher Scientific) mounting media and all slides were sealed. All slides were imaged analysed using a Nikon TI Eclipse confocal microscope (Nikon, Melville, NY, USA).

## 4 Statistical Analysis

Normality was tested using Shapiro–Wilks and Levene's test and visualized with a Q‐Q plot. Body weight, food intake, water intake, wheel activity and exercise distance were measured via repeated measures (RM) ANOVA. Based on normality results, bone and muscle outcomes were analysed with a two‐way ANOVA or Friedman test to determine differences between treatments. If omnibus significance was found, a post hoc analysis was completed using pairwise comparisons with a Sidak correction. Significance was considered at *p* < 0.05. Data are expressed in figures with mean ± SD with individual data points. Statistical analysis and visualization were performed with Graph Pad Prism v10.1.2.

## 5 Results

**FIGURE 1:**
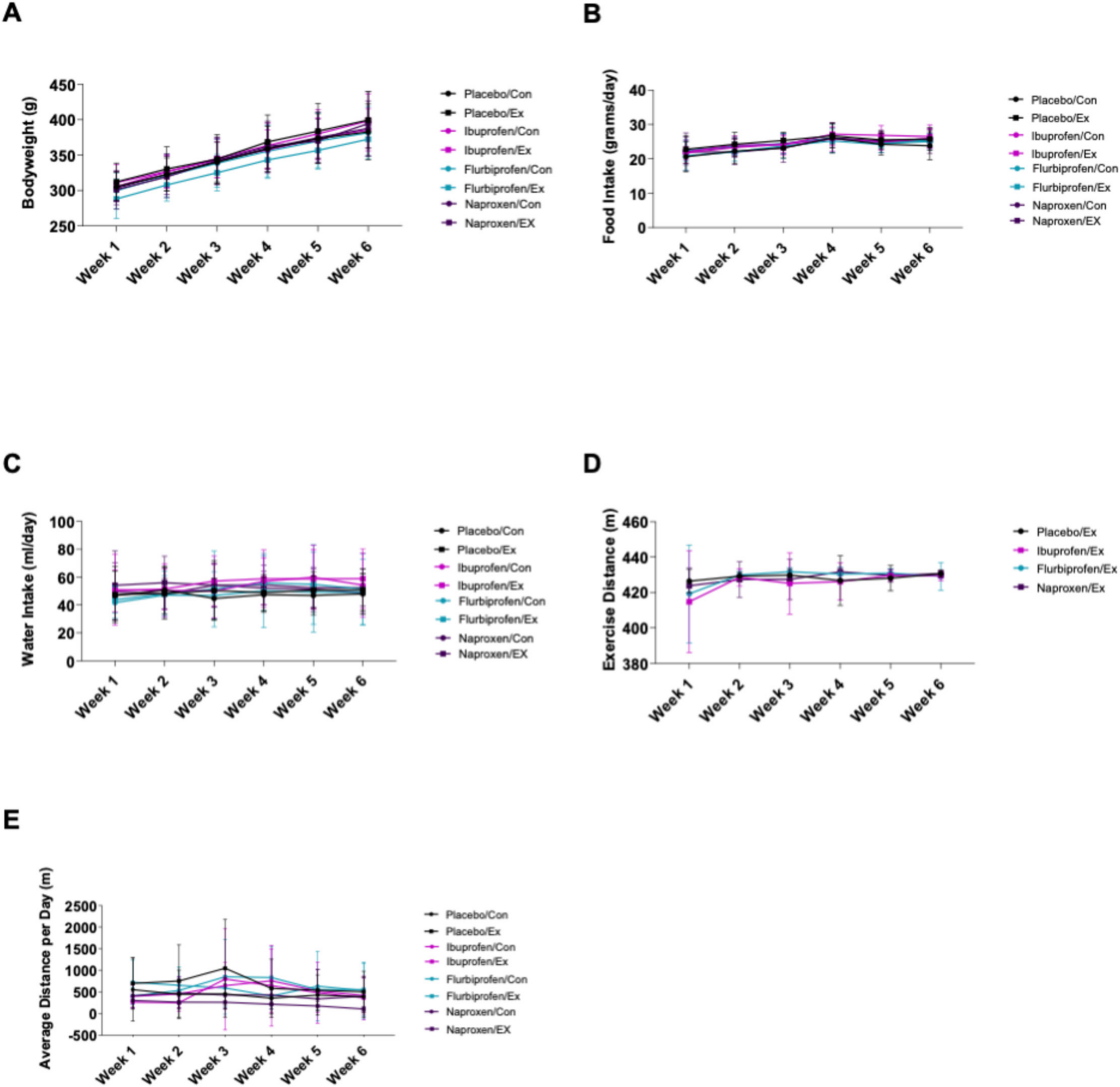
Bodyweight, food and water intake. Daily (A) bodyweight, (B) food intake, (C) water intake, (D) exercise distance and (E) wheel cage activity (E) were quantified over the course of 6 weeks. Data are representative of *n* = 8–10 per group and expressed as mean ± SD.

Throughout the results, treatment refers to placebo, ibuprofen, naproxen sodium or flurbiprofen; condition refers to exercise or control; and time refers to the week data was collected (i.e., Week 1). There was no treatment by condition effect for body weight (*p* = 0.815), but there was a time effect (*p* < 0.0001, Figure 1A) where all groups increased body weight over time. There was also no treatment by condition effect for food intake (*p* = 0.993) although there was a time effect (*p* < 0.0001, Figure 1B) with all groups increasing food intake over time. Similarly, there was no treatment by condition effect for water intake (*p* = 0.987, Figure 1C), but again, there was a time effect (*p* = 0.038, Figure 1C). There was no significant treatment by group effect for distance in groups who exercised (*p* = 0.898) although there was a time effect (*p* = 0.002, Figure 1D) likely due to the animals becoming more comfortable after the first week on the treadmill. There was no treatment by condition effect for wheel running activity (*p* = 0.99, Figure 1E) or the number of stimulations or total time of stimulations in the exercise groups (Table S1, *p* > 0.05). We also found no treatment by condition effect for change in lean mass or fat mass (Figure S4, *p* > 0.05).

**FIGURE 2:**
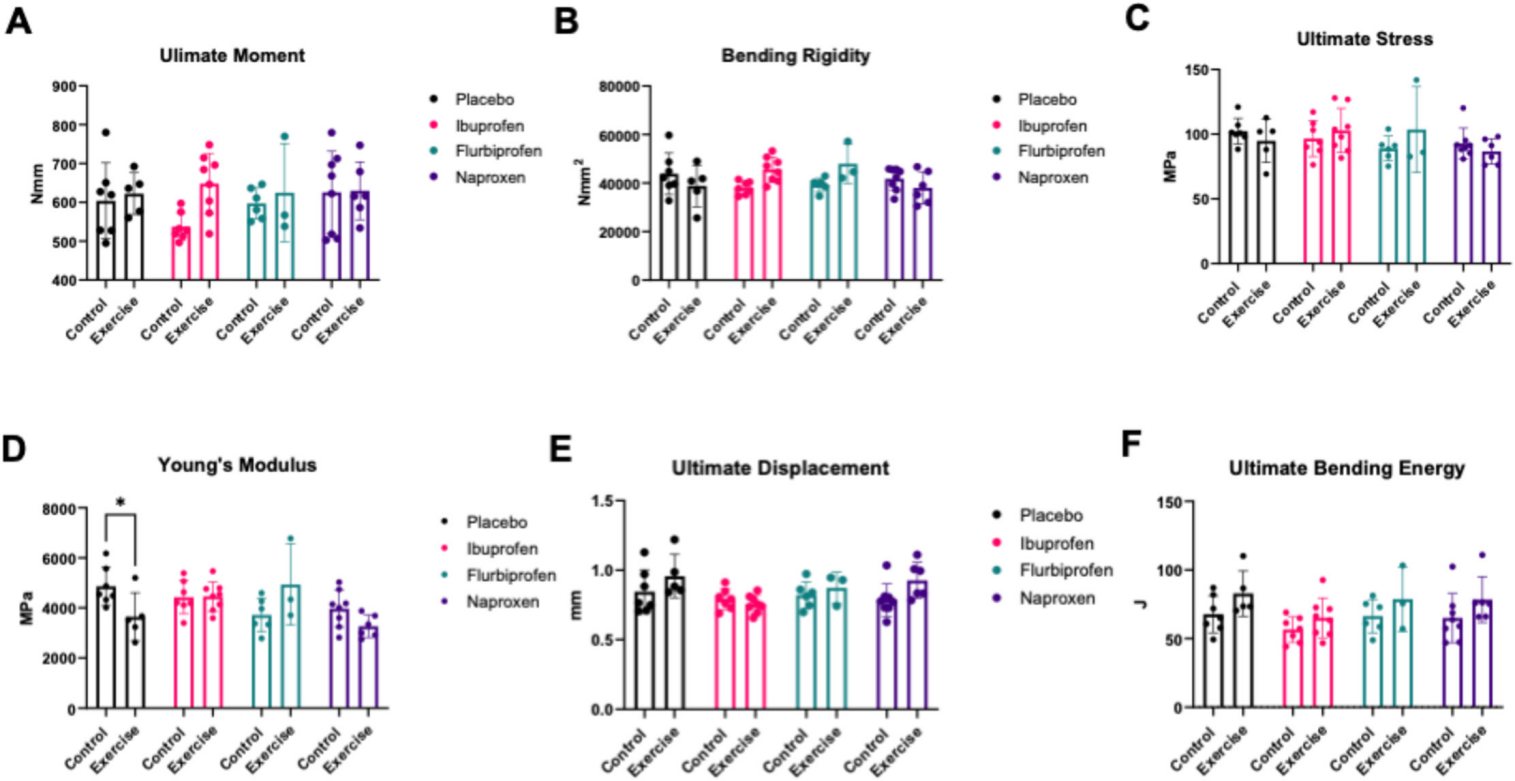
Three‐point bending in the femur. Effects of NSAIDs on femur structural and mechanical properties. Structural and mechanical properties were measured via a three‐point bending test. Femurs were placed with the femoral condyles facing down and a monotonic displacement ramp and 0.1 mm/s was applied until failure. The following measurements were obtained: (A) ultimate moment, (B) bending rigidity, (C) ultimate stress, (D) Young's modulus, (E) ultimate displacement and (F) ultimate bending energy. Data are representative of *n* = 4–8 per group and expressed as mean ± SD (*p < 0.05).

For 3 PB, there was no treatment by condition effect for ultimate moment (*p* = 0.288, Figure 2A). There was a significant treatment by condition effect for bending rigidity (*p* = 0.01, Figure 2B); however, there were no significant differences after a Sidak correction for multiple comparisons. There was no treatment by condition effect for ultimate stress (*p* = 0.282, Figure 2C). Although there was a significant treatment by exercise effect for Young's Modulus (*p* = 0.007, Figure 2D), where placebo control and placebo exercise were significantly different (*p* = 0.039). There was no treatment by condition effect for ultimate displacement (*p* = 0.194, Figure 2E). There was also no treatment by condition effect for ultimate bending energy (*p* = 0.949) although there was a significant exercise effect (*p* = 0.008, Figure 2F). There was no treatment by condition effect for postyield energy (*p* = 0.893), but there was an exercise effect (*p* = 0.019, Figure S1A). When measuring toughness, there was no treatment by condition effect (*p* = 0.654), but there was a significant exercise effect (*p* = 0.016, Figure S1B). Similarly, there was no treatment by condition effect for postyield toughness (*p* = 0.557), but there was a significant exercise effect (*p* = 0.022, Figure S1C). There was no treatment by condition effect for postyield displacement (*p* = 0.414), but there was a significant exercise (*p* = 0.041) and treatment effect (*p* = 0.022, Figure S1D). There was no treatment by time effect for ultimate strain (*p* = 0.358); however, there was a significant exercise effect (*p* = 0.018, Figure S1E). Finally, there was no treatment by condition effect for postyield strain (*p* = 0.489), with a significant exercise (*p* = 0.022) and treatment effect (*p* = 0.029, Figure S1F), but no comparisons were significant after a Sidak correction.

**FIGURE 3:**
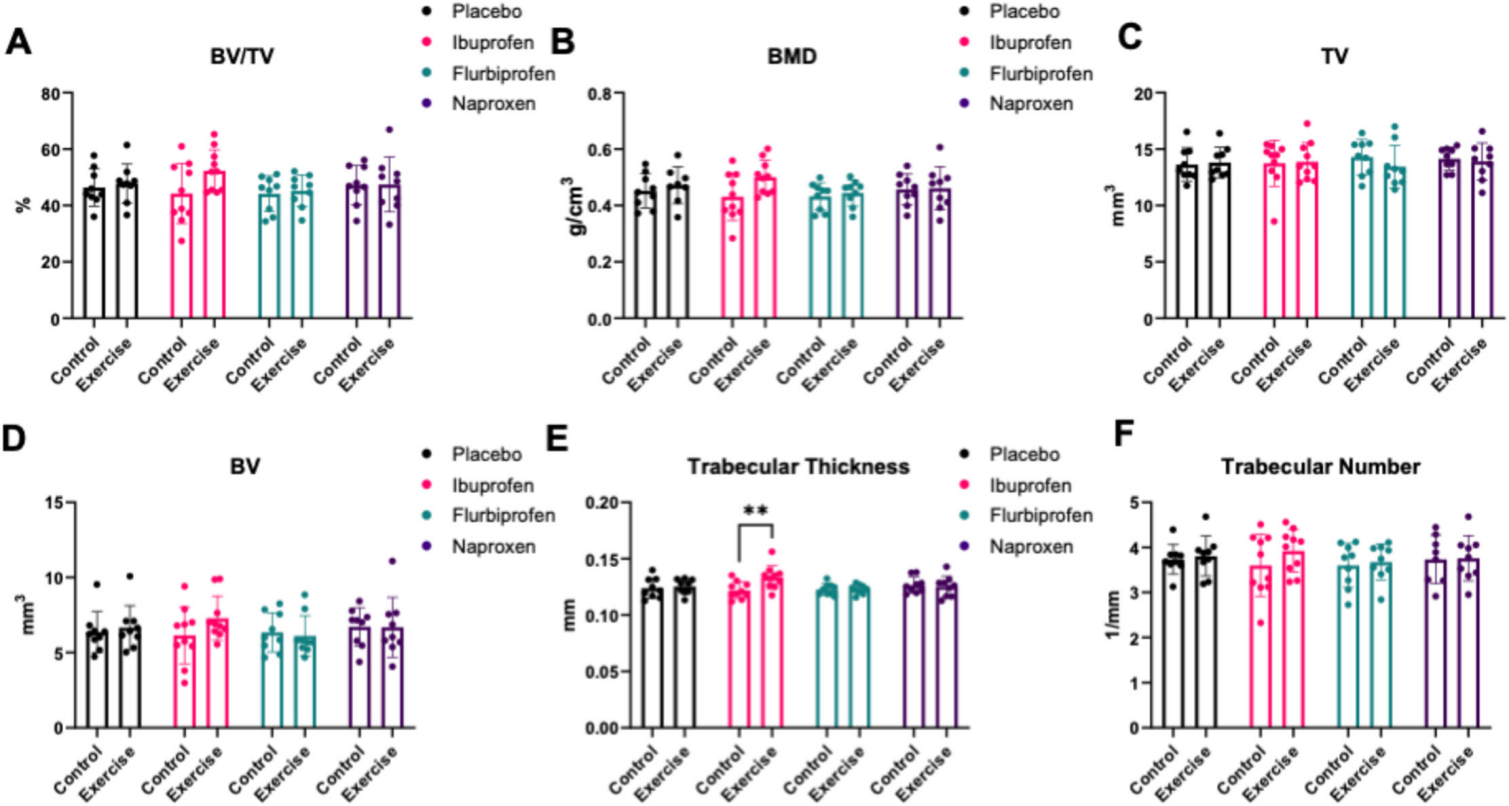
Trabecular geometry via microCT in the femur. Effects of NSAIDs on femur trabecular bone mass and geometry. Transverse femur slices were obtained using a Bruker Skyscan 1275 microCT utilizing a 1‐mm aluminium filter using 55‐kV and 181‐μA scan setting with 74 ms of exposure time to obtain measurements for (A) bone volume/trabecular volume (BV/TV), (B) bone mineral density (BMD), (C) tissue volume (TV), (D) bone volume (BV), (E) trabecular thickness and (F) trabecular number. Data are representative of *n* = 8–10 per group and expressed as mean ± SD (*p < 0.05).

MicroCT results were similar to 3 PB as there was minimal treatment by condition effects. Specifically, in the trabecular geometry of the femur, there were no treatment by condition effects for bone volume (BV)/tissue volume (TV) (*p* = 0.370, Figure 3A), bone mineral density (BMD) (*p* = 0.377, Figure 3B), tissue volume (*p* = 0.756, Figure 3C) and bone volume (*p* = 0.519, Figure 3D). Interestingly, there was a treatment by condition effect for trabecular thickness (*p* = 0.047, Figure 3E) where the ibuprofen plus exercise group increased trabecular thickness compared with the ibuprofen control group (*p* = 0.027). There was no treatment by condition effect for trabecular number (*p* = 0.786, Figure 3F), trabecular spacing (*p* = 0.552, Figure S2A), trabecular bone surface (BS)/TV (*p* = 0.831, Figure S2B) or trabecular BS/BV (*p* = 0.118, Figure S2C).

**FIGURE 4:**
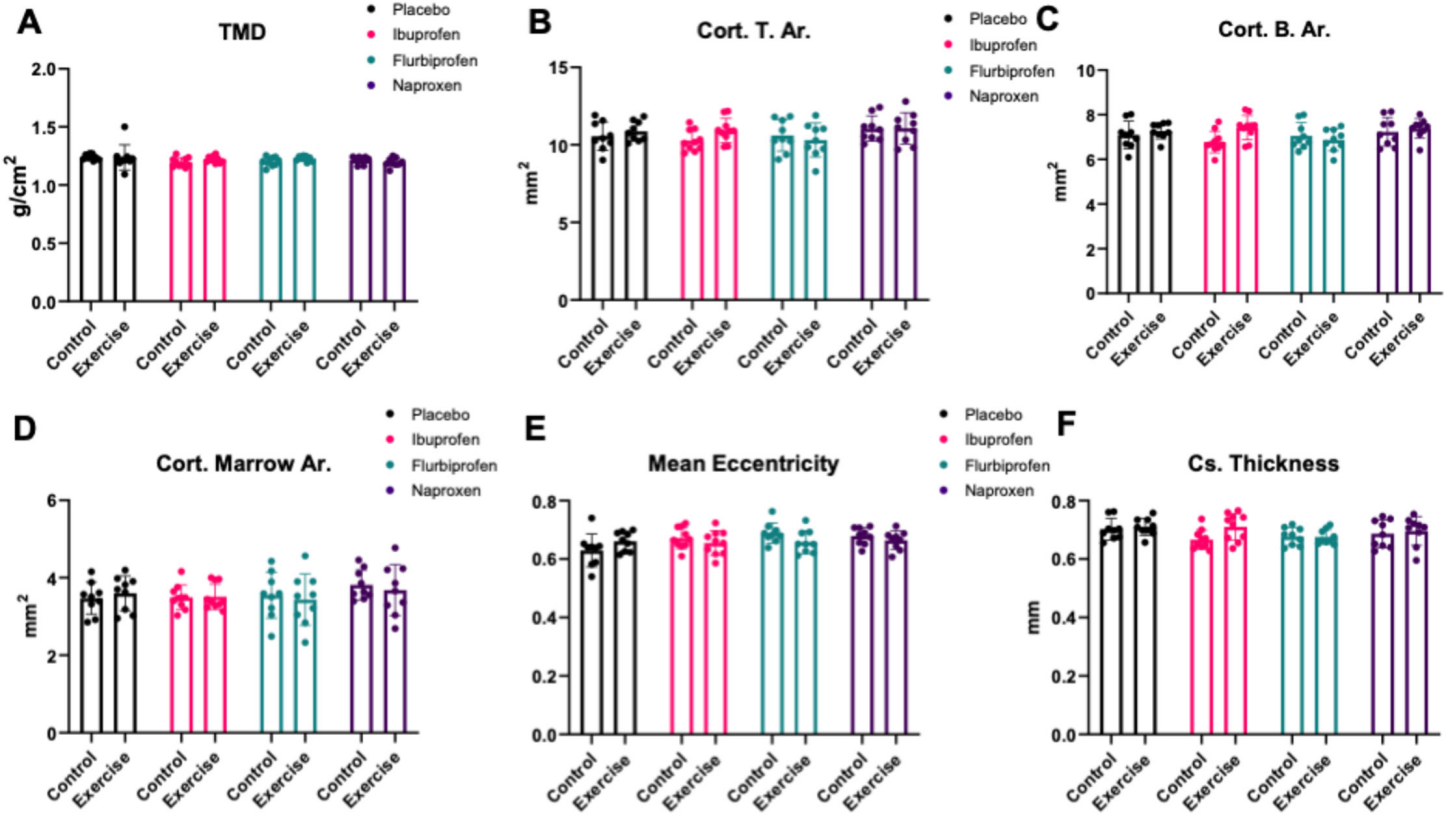
Cortical geometry via microCT in the femur. Effects of NSAIDs on femur cortical bone mass and geometry. Transverse femur slices were obtained using a Bruker Skyscan 1275 microCT utilizing a 1‐mm aluminium filter using 55‐kV and 181‐μA scan setting with 74 ms of exposure time to obtain measurements for (A) tissue mineral density (TMD), (B) cortical tissue area (Cort. T. Ar.), (C) cortical bone area (Cort. B. Ar.), (D) cortical marrow area (Cort. Marrow Ar.), (E) mean eccentricity, (F) cortical thickness (cs. thickness). Data are representative of *n* = 8–10 per group and expressed as mean ± SD (*p < 0.05).

For cortical geometry of the femur, there were no treatment by condition effects for cortical tissue mineral density (TMD, *p* = 0.406, Figure 4A), cortical tissue area (Cort. T. Ar., *p* = 0.389, Figure 4B), cortical bone area (Cort. B. Ar, *p* = 0.124, Figure 4C), cortical marrow area (Cort. Marrow. Ar, *p* = 0.831, Figure 4D), mean eccentricity (*p* = 0.092, Figure 4E), cross‐sectional thickness (Cs. Thickness, *p* = 0.279, Figure 4F), cortical mean polar moment of inertia (MMI, *p* = 0.272, Figure S3A) and Cort. B. Ar/T. Ar. (*p* = 0.501, Figure S3B).

**FIGURE 5:**
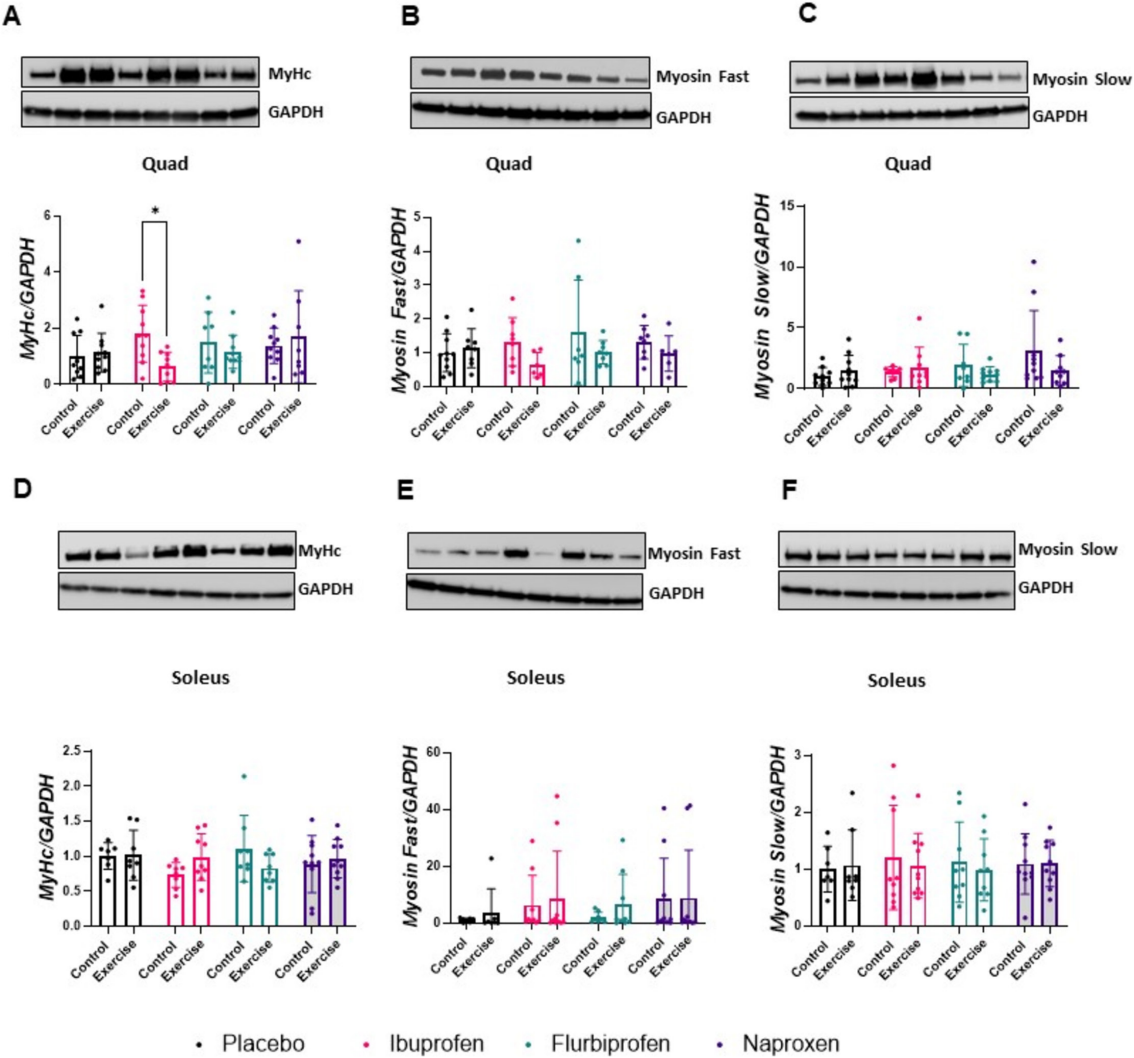
Myosin content in the quadriceps and soleus. Effects of NSAIDs on myosin heavy chain protein abundance. Western immunoblotting was performed using protein lysates using the following antibodies: (A) quadriceps myosin heavy chain (MyHc), (B) quadriceps MyHc slow and (C) quadriceps MyHc fast, (D) soleus MyHC, (E) soleus MyHc fast (F) and soleus MyHc slow. Representative western blot images are depicted above their respective graphs. Results were all first normalized to GAPDH and then expressed relative to placebo control which is set to 1.0. Data are representative of *n* = 6–9 per group and expressed as mean ± SD (*p < 0.05).

For MyHC in the quadriceps, we found a treatment by condition effect (*p* = 0.046) and a significant difference between ibuprofen control and ibuprofen exercise for total myosin heavy chain (*p* = 0.016, Figure 5A). However, there was no treatment by condition effect for myosin fast (*p* = 0.383, Figure 5B) or myosin slow (*p* = 0.179, Figure 5C). In the soleus, there was no treatment by condition effect for total myosin heavy chain (*p* = 0.116, Figure 5D), myosin fast (*p* = 0.959, Figure 5E) or myosin slow (*p* = 0.9364, Figure 5F). Representative immunoblot images can be seen in the figures.

**FIGURE 6:**
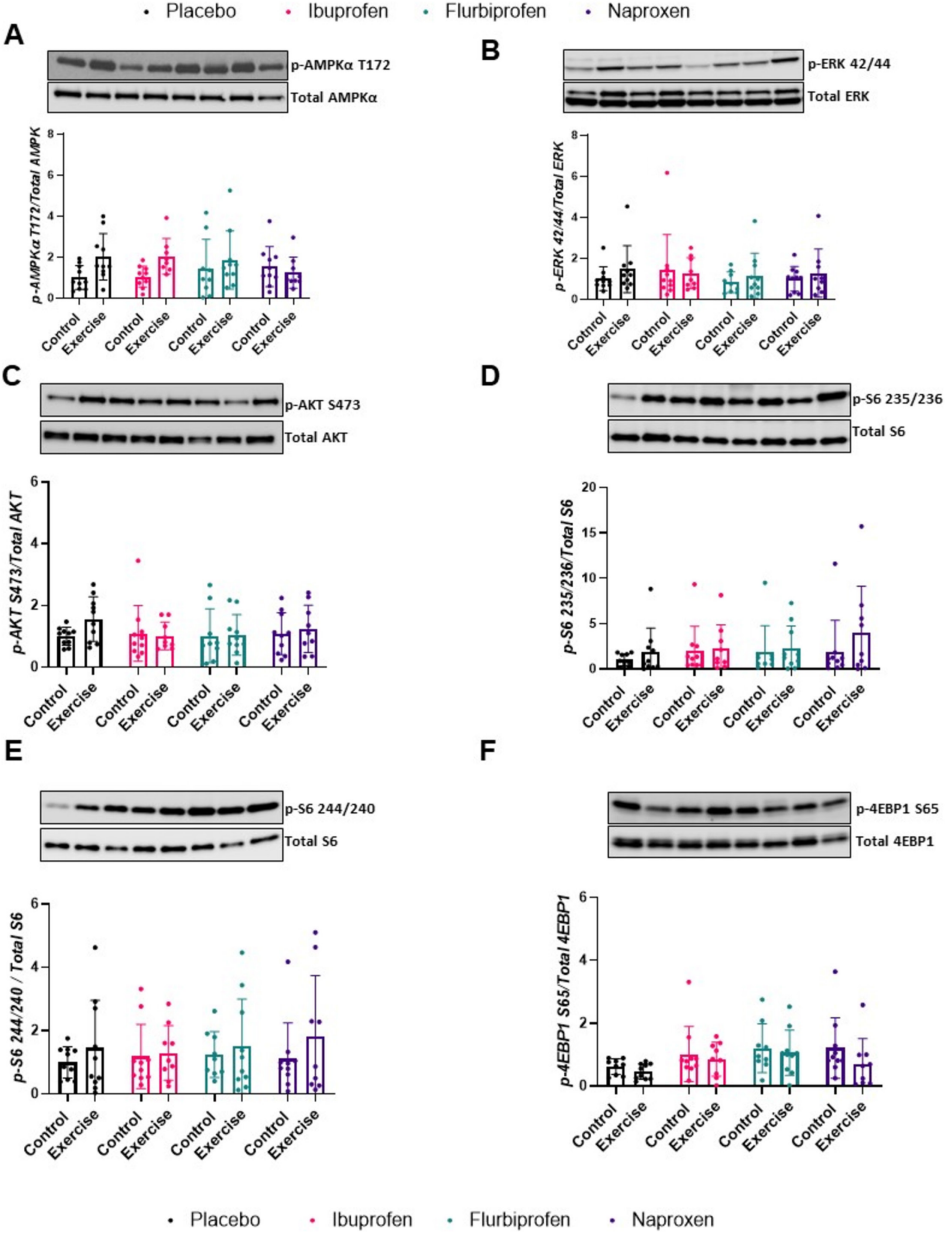
Quadriceps muscle signalling. Effects of NSAIDs on signalling pathway proteins in quadricep muscle. Western immunoblotting was performed using protein lysates using the following antibodies: (A) phospho‐AMPKα Thr172, (B) phospho‐ERK 42/44, (C) phospho‐AKT Ser473, (D) phospho‐S6 Ser235/236, (E) phospho‐S6 Ser240/244 and (F) p‐4EBP1 s65. Representative western blot images are depicted above their respective graphs. Results were all first normalized to GAPDH or the corresponding total protein then expressed relative to placebo control, which is set to 1.0. Data are representative of *n* = 6–9 per group and expressed as mean ± SD (*p < 0.05).

When characterizing the molecular signalling pathways in the quadriceps postexercise, there was no treatment by condition effect for p‐AMPKT172/Total AMPK (*p* = 0.085) but there was an exercise effect (*p* = 0.027, Figure 6A). There was no treatment by condition effect for p‐ERK42/44/Total ERK (*p* = 0.792, Figure 6B), p‐AKTS473/Total AKT (*p* = 0.496, Figure 6C), (*p* = 0.776, Figure 6D) or p‐S6244/244/Total S6 (*p* = 0.886, Figure 6E). Finally, there was no treatment by condition effect for p‐4EBP1/Total 4EBP1 (*p* = 0.709), but there was an exercise effect (*p* = 0.035, Figure 6F).

**FIGURE 7:**
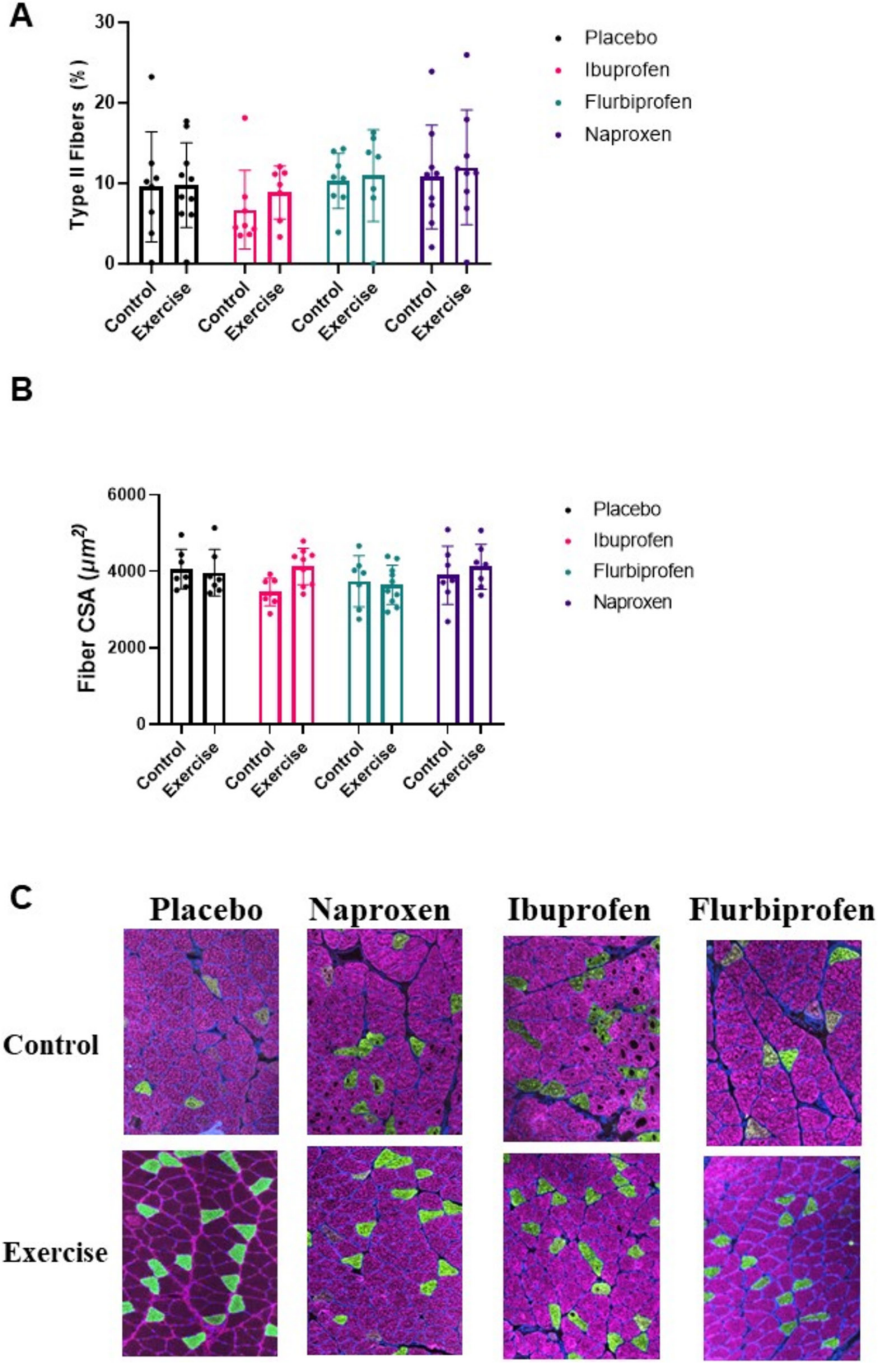
Soleus muscle fibre histology. Soleus were sliced using a microtome to a thickness of 6–8 μM and mounted on slides and stained for Type I fibres, Type II fibres and laminin. (A) Fibre type percentage, (B) muscle fibre cross‐sectional area and (C) representative images of muscle histology.

When analysing muscle fibre type in the soleus, there was no significant treatment by condition effect for fibre type percentage (*p* = 0.967, Figure 7A) or fibre CSA (*p* = 0.652, Figure 7B). Representative images can be seen in Figure 7C.

## 6 Discussion

This study investigated how 6 weeks of daily naproxen sodium, flurbiprofen, ibuprofen or a placebo influenced skeletal muscle and bone adaptations to treadmill exercise in male rats. Unlike previous studies that have used supraphysiological doses or extreme models, our approach focused on testing NSAID doses below the NOAEL for rodents and a dose that humans may consume before or after exercise, closely mirroring physiologically relevant conditions. Our results show that although treadmill exercise promotes musculoskeletal adaptations, NSAIDs do not considerably impact these adaptations in a positive or negative manner. This suggests that prior reports of inhibitory effects on musculoskeletal adaptation may be attributed to higher‐than‐physiological NSAID doses or extreme experimental models.

Bone is a mechanoadaptive tissue with the osteocytes, osteoblasts and osteoclasts working together to detect changes in the mechanical loading environment and address areas of microdamage accrual [24]. Osteocytes, in a process termed targeted remodelling, can signal osteoclasts (bone resorption cells) to remove the areas of damage and bone can subsequently replace damaged bone tissue with healthy new tissue via osteoblasts (bone formation cells). This remodelling cycle can lead to temporary porosity and decrements in the structural integrity of bone [13]. In adaptive bone formation, osteocytes sense mechanical loading and signal osteoblasts, independent of osteoclastic bone resorption, in order to form new bone, often on the periosteal surfaces [13]. Even modest amounts of this type of bone formation greatly increase the fatigue resistance of bone. NSAIDs have been shown to have adverse effects on osteoblast‐mediated bone formation in vitro [25]. Suppression of adaptive bone formation with NSAID use, likely due to the inhibition of prostaglandin formation during mechanical loading, has been experimentally demonstrated in animals and may underpin increased stress fracture risk in humans [20, 26, 27, 28, 29].

Previous experiments have found that NSAIDs reduce bone formation in response to a single bout of mechanical loading in rodents [26, 27, 29] but not humans [30]. For example, naproxen sodium has been shown to inhibit bone formation in response to six bouts of forelimb loading over 2 weeks [20]. Conversely, flurbiprofen—a strong COX‐1 inhibitor—has demonstrated positive effects on bone formation in rats when administered over 21 days using a microdosing approach (0.5 mg/kg/day) [31]. Herein, we opted for a moderate running exercise that reflects the current American College of Sports Medicine exercise guidelines consisting of ~150 min of moderate‐intensity aerobic split over 5 days per week [32]. Interestingly, we observed Young's Modulus was significantly diminished (−25%) with exercise in the placebo group, but not in the NSAID groups, which is similar to one other study [33]. Since we did not observe changes in BMD, cross‐sectional geometry or bending rigidity in response to NSAIDs, our findings suggest that NSAIDs may counteract other exercise‐induced changes that affect Young's Modulus, such as porosity.

We also observed increased trabecular thickness in the ibuprofen exercise group compared with the ibuprofen control group. This may indicate ibuprofen is beneficial to bone adaptations even though these findings did not translate to mechanical bone properties. Outside of those adaptations, there were minimal differences in bone mass, geometry and both structural and mechanical properties between treatments. Our results are consistent with a study closely resembling ours in design, but with a much lower ibuprofen dosage (30 vs. 200 mg/kg/day in our study) reporting no impact on bone adaptations following 12 weeks of treadmill running, as assessed through microCT and 3 PB, suggesting that very low doses of ibuprofen also do not impact bone [34]. Another study by Jain et al., which combined a high‐repetition/high‐force task model with ibuprofen treatment (45 mg/kg), found reductions in osteoclast response and no changes in osteoblast response, indicating that ibuprofen may be maladaptive by preventing bone remodelling [35]. The disparity between studies is likely related to the exercise stimulus. In our study, we used a stimulus that was meant to mimic low‐intensity exercise, similar to what occurs in humans with aerobic‐based exercise, rather than the high‐repetition or high‐force stimulus used by Jain et al. [35]. Although our study demonstrated several exercise‐induced effects on skeletal tissue, they were of comparably smaller magnitude than those seen in mechanically induced studies, and our experiments provide important data to demonstrate that prolonged NSAID consumption in combination with treadmill exercise does not negatively affect bone.

To determine how skeletal muscle adapts to treadmill running with and without NSAIDs, we measured protein abundance, phosphorylation status, muscle fibre type and muscle fibre cross‐sectional area (CSA). When analysing protein abundance of myosin heavy chain, we first used an antibody that measured all myosin heavy chain isoforms and then analysed the fast and slow isoforms, independently, to determine if there were any isoform‐specific effects. We did not find any NSAID or exercise effects, except in the quadriceps, where the ibuprofen control group had more myosin‐heavy chain than ibuprofen exercise, which appears to be driven by a reduction in the myosin slow isoform. This could indicate a potentially detrimental effect on muscle turnover that leads to reduced muscle function with a longer term exercise intervention. The effect was only seen in the ibuprofen group, likely due to the NSAID inhibiting both COX‐1 and COX‐2 isoforms instead of having a strong affinity for a single COX isoform. These findings were not replicated in the soleus, potentially due to fibre type differences (i.e., soleus is > 85% myosin slow) or muscle activity involved in treadmill running, which aligns with a study completed in mice that analysed the plantaris muscle [10].

Several studies have also measured the effects of NSAIDs on skeletal muscle in rodents [7, 9, 10, 36]. Bondesen et al. treated mice with a COX‐1 (SC‐560, 3 mg/kg/day) and a COX‐2 (SC‐256, 6 mg/kg/day) inhibitor in drinking water prior to an extreme muscle injury induced by local freeze damage and found that COX‐2, but not COX‐1, was required for normal muscle regeneration [7]. Novak et al. found that NS‐398, a strong COX‐2 inhibitor, blunted increases in muscle growth, suggesting that COX‐2 is necessary for muscle hypertrophy [36]. Soltow et al. used a synergistic ablation model to induce overload of the plantaris muscle, which was combined with ibuprofen treatment (20 mg/kg/day), and found that ibuprofen caused a significant reduction (~50%) of muscle hypertrophy after 14 days although they reported no effect of ibuprofen on COX mRNA [9]. These three studies suggest that COX‐2 is required for muscle growth and regeneration in extreme situations, which may not translate to humans. In a more practical study, with a similar design to ours, ibuprofen did not influence running distance–dependent adaptations in the skeletal muscle of mice, which is in agreement with our findings [10]. Taken together, NSAIDs may only influence skeletal muscle growth and regeneration in extreme models, such as a genetic knockout or synergistic ablation, and NSAIDs likely influence adaptations to resistance exercise differently than treadmill training.

NSAIDs affect translational signalling and muscle protein synthesis in humans [37, 38, 39]. For example, Markworth et al. [37] observed a decrease in p‐RPS6 (Ser235/S36 and Ser240/244) after resistance exercise with ibuprofen. Although our experiments are not directly comparable with these, we found no differences between groups for p‐RPS6 or p‐4EBP1. We took a broad approach when probing pathways, opting to measure p‐AMPK and p‐ERK given previous detrimental effects found in cell culture models [11, 40]. To further characterize the chronic skeletal muscle response, we measured the muscle fibre CSA and fibre type of the soleus and found no differences amongst the treatment groups, probably due to the endurance‐based exercise and moderate NSAID dosage. Taken together, our data in the quadriceps and soleus indicate NSAIDs had minimal impact on muscle signalling with 6 weeks of combined training and NSAID treatment.

Our study has several strengths. First, we comprehensively quantified both muscle and bone adaptations to NSAID treatment with treadmill training. In doing this, we simultaneously compared the effects of NSAIDs with a wider range of COX selectivity than any study ever published. We also used multiple muscle types, including the quadriceps and soleus, which vary in their typical fibre type distribution to better characterize potential changes. Although prior studies have provided drugs through drinking water [7, 9, 10], this method of administration has the limitation of not being able to precisely control the timing and exact amount of drug provided to each animal, and we chose this method to better compare with the literature. Another limitation that could be argued is the exercise modality and intensity used in our study. Particularly for a response in bone, a higher intensity modality may need to find NSAID‐specific effects. Our goal was to mimic low‐intensity exercise, similar to how many humans engage in endurance exercise programs (e.g., brisk walking or jogging) combined with prolonged NSAID consumption. We also did not measure NSAID concentrations in the blood or muscle due to a lack of access to LC/MS equipment. Another limitation of this study is the absence of COX‐1 and COX‐2 measurements, as these assessments are challenging and often do not reliably reflect NSAID usage, particularly in skeletal muscle, without incubation in high concentrations of AA. Additionally, previous studies to which we directly compare our results have not observed changes in COX‐1/2 activity for similar reasons [9, 10, 26, 36]. We further recognize that our findings may not fully capture the potential influence of NSAIDs on musculoskeletal adaptations in females. Sex‐specific hormonal profiles, particularly oestrogen, have been shown to affect bone density, muscle mass and responses to both exercise and pharmacological agents like NSAIDs. Additionally, females may exhibit different inflammatory and oxidative stress responses to exercise, which could modulate the effects of NSAIDs on muscle signalling pathways. Higher NSAID dosages, akin to those recommended for postoperative care, or patients on prescriptions for pain should be tested to verify whether the outcomes observed in our study persist across different conditions. Investigations should also extend to other tissues, such as tendons and ligaments to provide a more comprehensive view. In conclusion, our research suggests that NSAIDs exert minimal influence on musculoskeletal adaptations when combined with treadmill exercise in male rats.

## Disclosure

The opinions or assertions contained herein are the private views of the authors and are not to be construed as official or reflecting the views of the Army or the Department of Defense. Any citations of commercial organizations and trade names in this report do not constitute an official Department of the Army endorsement of approval of the products or services of these organizations.

## Conflicts of Interest

The authors declare no conflicts of interest.

## Supporting information


**Figure S1** Trabecular geometry via microCT in the femur. Effects of NSAIDs on femur trabecular bone mass and geometry. (A) Postyield energy (TV), (B) toughness, (C) postyield toughness, (D) postyield displacement, (E) ultimate strain and (F) postyield strain. Data are representative of *n* = 8–10 per group and expressed as mean ± SD (*p < 0.05).

**Figure S2** Cortical geometry via microCT in the femur. Effects of NSAIDs on femur cortical bone mass and geometry. Transverse femur slices were obtained using a Bruker Skyscan 1275 microCT utilizing a 1‐mm aluminium filter using 55‐kV and 181‐μA scan setting with 74 ms of exposure time to obtain measurements for (A) trabecular spacing, (B) trabecular BS/TV and (C) trabecular BS/BV. Data are representative of *n* = 8–10 per group and expressed as mean ± SD (*p < 0.05).

**Figure S3** Cortical geometry via microCT in the femur. Effects of NSAIDs on femur cortical bone mass and geometry. Transverse femur slices were obtained using a Bruker Skyscan 1275 microCT utilizing a 1‐mm aluminium filter using 55‐kV and 181‐μA scan setting with 74 ms of exposure time to obtain measurements for (A) mean polar moment of inertia (MMI) and (B) cortical bone area/trabecular area (cort. B. Ar./T. Ar.). Data are representative of *n* = 8–10 per group and expressed as mean ± SD (*p < 0.05).

**Figure S4** Nuclear magnetic resonance imaging. Change in (A) body fat percent change and (B) lean mass during the study.

**Table S1** Average absolute number of stimulations and time of stimulations (seconds) administered via electrical shock pad (> 0.2 mA) per day/bout of exercise. Only exercise groups received stimulations.

## Data Availability

The data that support the findings of this study are available on request from the corresponding author. The data are not publicly available because of data sharing agreements required by the author's organization.
