# Supplementary figures and images for: Ibuprofen, Flurbiprofen or Naproxen Sodium Minimally Influences Musculoskeletal Adaptations to Treadmill Exercise in Rats
Source: J Cachexia Sarcopenia Muscle. 2025 Apr 10;16(2):e13798. doi: 10.1002/jcsm.13798 (PMC11985359; doi:10.1002/jcsm.13798)

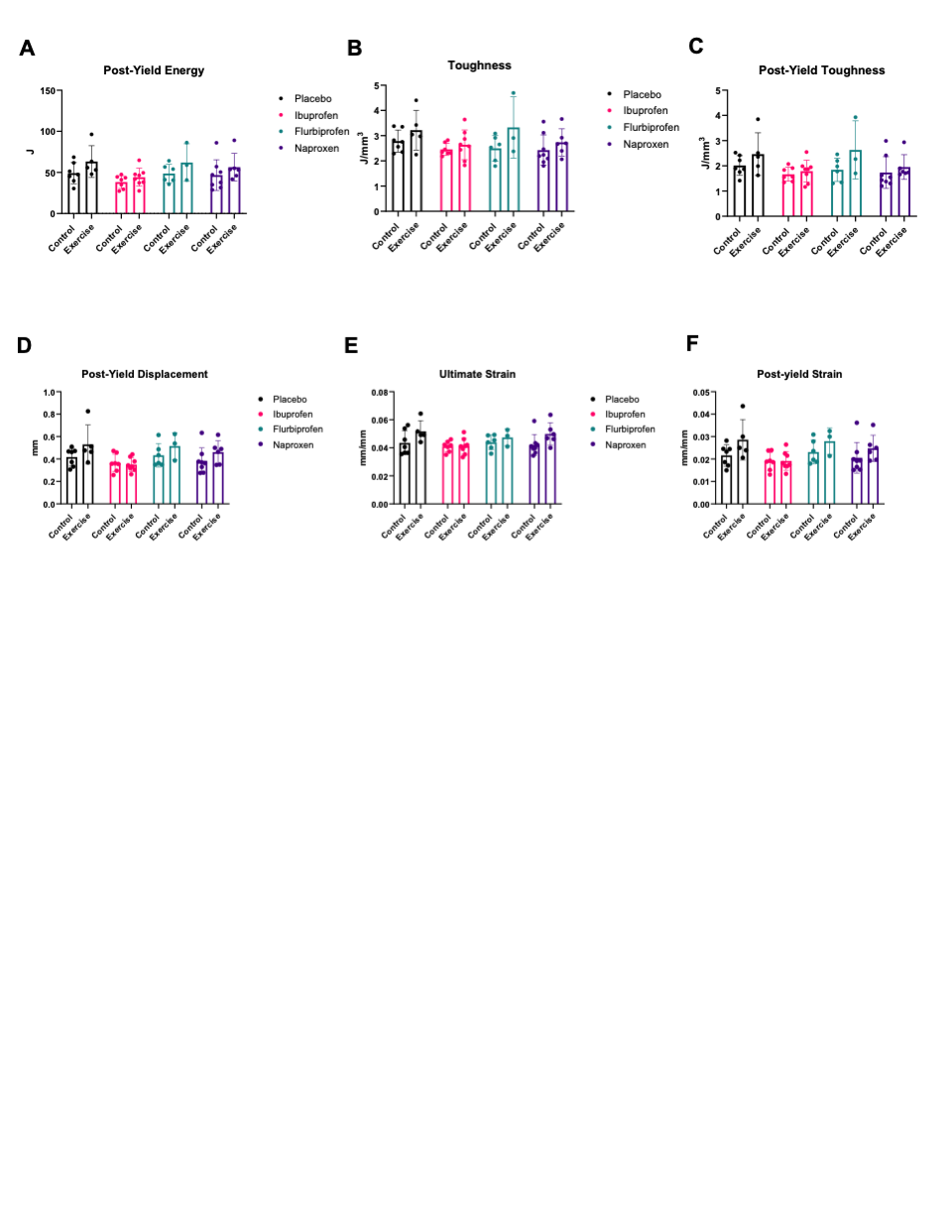

Supplement: Supplementary file 1 — Figure S1 Trabecular geometry via microCT in the femur. Effects of NSAIDs on femur trabecular bone mass and geometry. (A) Postyield energy (TV), (B) toughness, (C) postyield toughness, (D) postyield displacement, (E) ultimate strain and (F) postyield strain. Data are representative of n = 8–10 per group and expressed as mean ± SD (*p < 0.05). [file JCSM-16-e13798-s001.png]

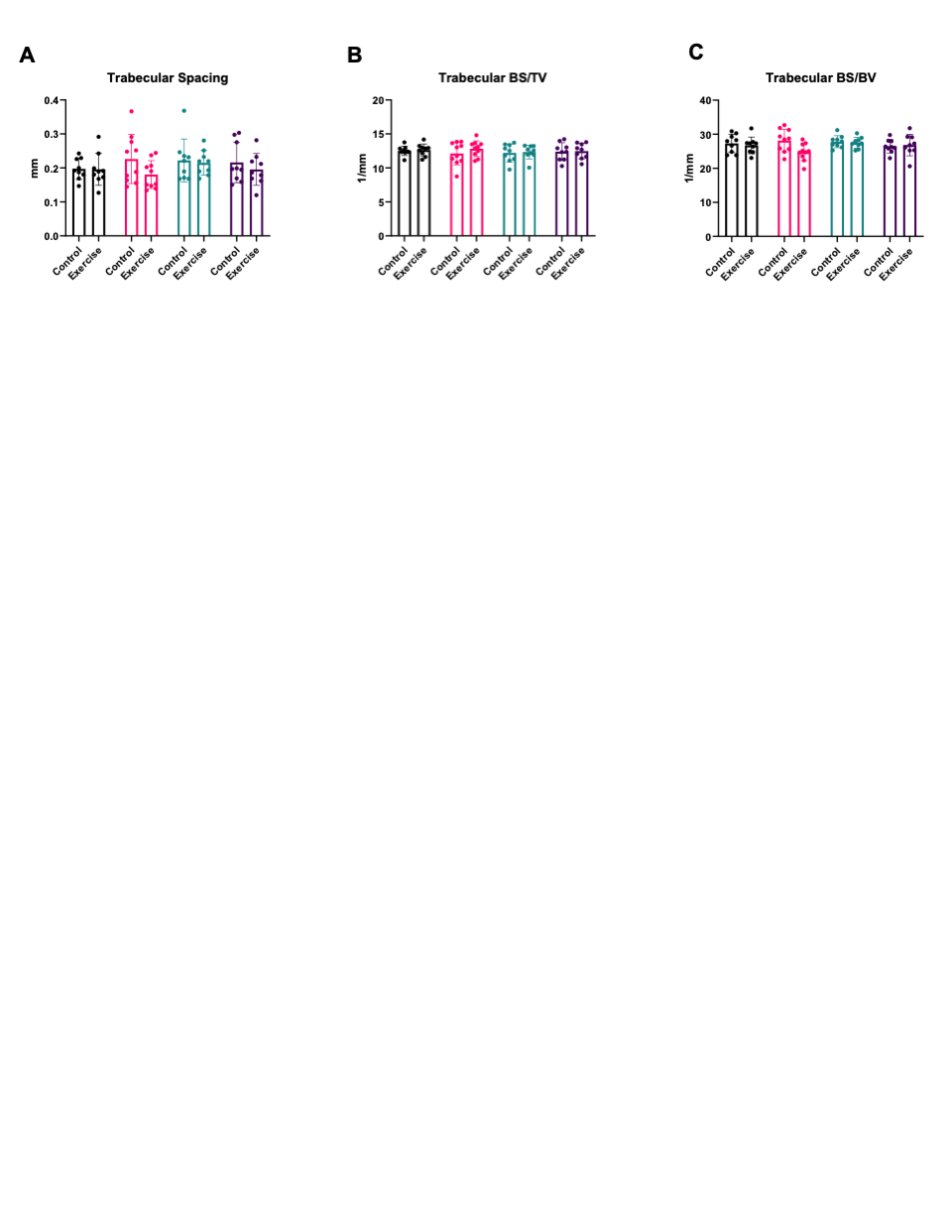

Supplement: Supplementary file 2 — Figure S2 Cortical geometry via microCT in the femur. Effects of NSAIDs on femur cortical bone mass and geometry. Transverse femur slices were obtained using a Bruker Skyscan 1275 microCT utilizing a 1‐mm aluminium filter using 55‐kV and 181‐μA scan setting with 74 ms of exposure time to obtain measurements for (A) trabecular spacing, (B) trabecular BS/TV and (C) trabecular BS/BV. Data are representative of n = 8–10 per group and expressed as mean ± SD (*p < 0.05). [file JCSM-16-e13798-s002.png]

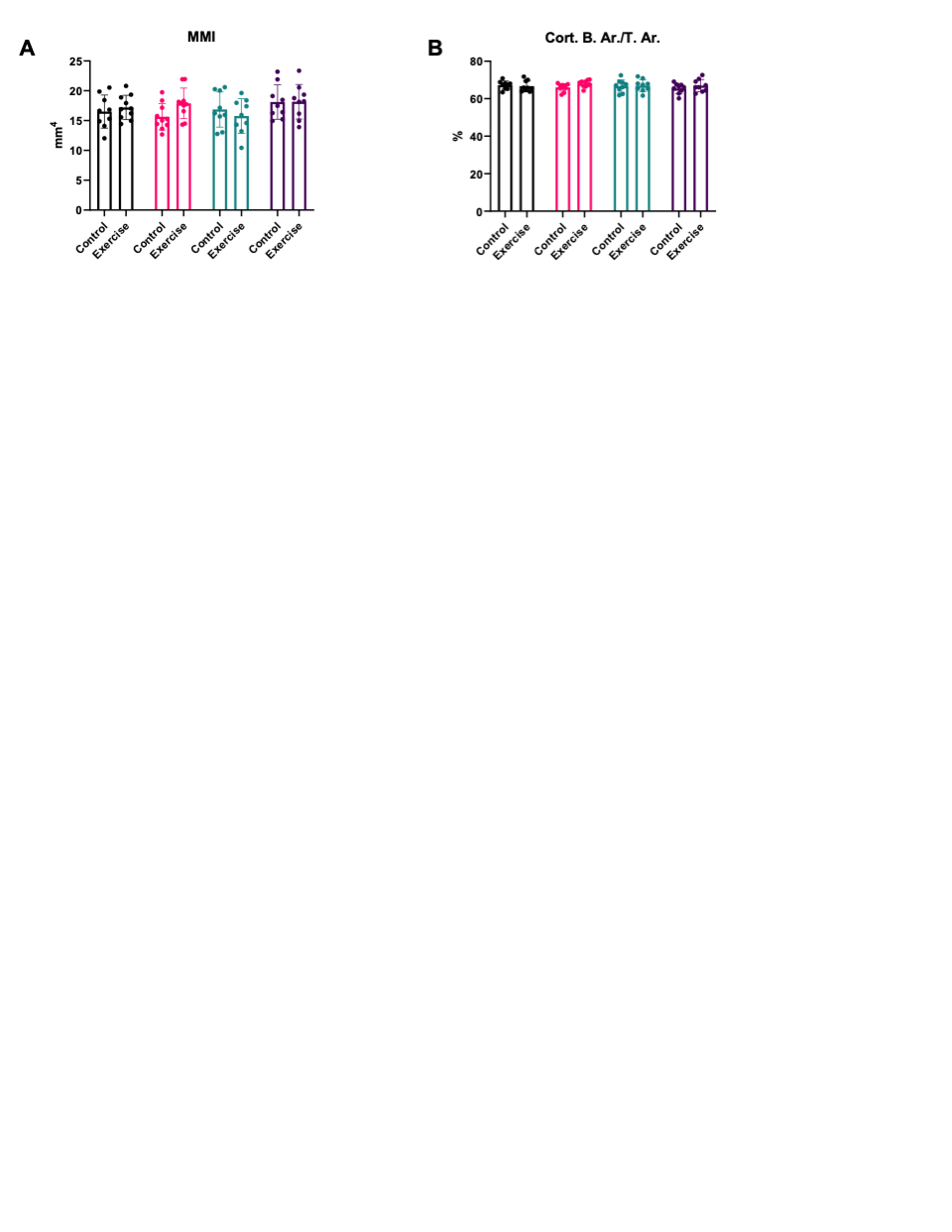

Supplement: Supplementary file 3 — Figure S3 Cortical geometry via microCT in the femur. Effects of NSAIDs on femur cortical bone mass and geometry. Transverse femur slices were obtained using a Bruker Skyscan 1275 microCT utilizing a 1‐mm aluminium filter using 55‐kV and 181‐μA scan setting with 74 ms of exposure time to obtain measurements for (A) mean polar moment of inertia (MMI) and (B) cortical bone area/trabecular area (cort. B. Ar./T. Ar.). Data are representative of n = 8–10 per group and expressed as mean ± SD (*p < 0.05). [file JCSM-16-e13798-s003.png]

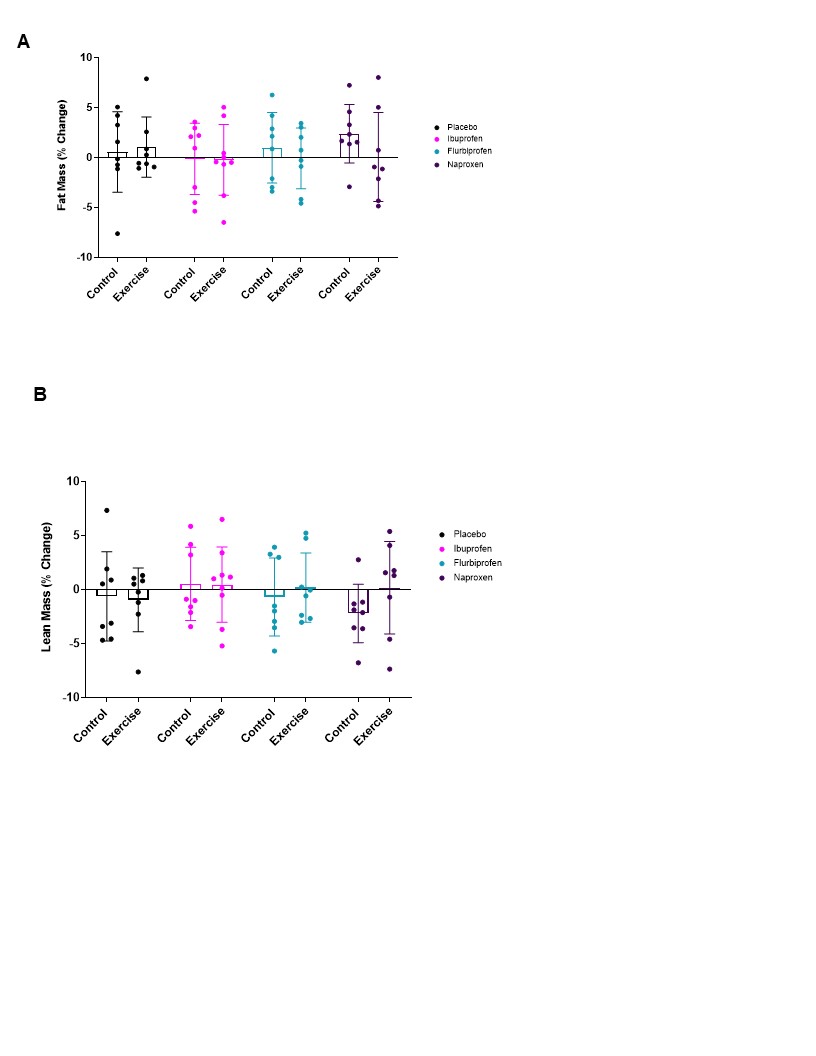

Supplement: Supplementary file 4 — Figure S4 Nuclear magnetic resonance imaging. Change in (A) body fat percent change and (B) lean mass during the study. [file JCSM-16-e13798-s005.jpg]
