# Supplementary material for: Ibuprofen, Flurbiprofen or Naproxen Sodium Minimally Influences Musculoskeletal Adaptations to Treadmill Exercise in Rats
Source: J Cachexia Sarcopenia Muscle. 2025 Apr 10;16(2):e13798. doi: 10.1002/jcsm.13798 (PMC11985359; doi:10.1002/jcsm.13798)
Supplement: Supplementary file 5 — Table S1 Average absolute number of stimulations and time of stimulations (seconds) administered via electrical shock pad (> 0.2 mA) per day/bout of exercise. Only exercise groups received stimulations. [file JCSM-16-e13798-s004.docx]

| **Treatment** |  | **Week 1** | **Week 2** | **Week 3** | **Week 4** | **Week 5** | **Week 6** |
| --- | --- | --- | --- | --- | --- | --- | --- |
| **Placebo** | Number of Stimulations | 28 ± 26 | 31 ± 32 | 18 ± 16 | 11 ± 9 | 8 ± 10 | 15 ± 8 |
|  | Total Stimulation Time (sec) | 11 ± 6 | 9 ± 10 | 9 ± 8 | 6 ± 9 | 5 ± 8 | 7 ± 8 |
| **Naproxen** | Number of Stimulations | 29 ± 21 | 13 ± 18 | 7 ± 6 | 7 ± 6 | 9 ± 6 | 15 ± 10 |
|  | Total Stimulation Time (sec) | 12 ± 10 | 9 ± 10 | 3 ± 3 | 3 ± 6 | 7 ± 11 | 9 ± 9 |
| **Ibuprofen** | Number of Stimulations | 46 ± 54 | 18 ± 15 | 18 ± 19 | 28 ± 34 | 26 ± 37 | 31 ± 48 |
|  | Total Stimulation Time (sec) | 11 ± 9 | 11 ± 8 | 5 ± 5 | 7 ± 7 | 7 ± 7 | 10 ± 12 |
| **Flurbiprofen** | Number of Stimulations | 28 ± 27 | 18 ± 11 | 9 ± 6 | 13 ± 10 | 9 ± 7 | 15 ± 14 |
|  | Total Stimulation Time (sec) | 10 ± 7 | 10 ± 9 | 5 ± 5 | 3 ± 2 | 2 ± 2 | 6 ± 7 |
